# Body image, eating distress and emotional-behavioral difficulties among adolescents in Mbarara, Southwestern Uganda

**DOI:** 10.1186/s12889-024-18973-1

**Published:** 2024-06-04

**Authors:** Joan Abaatyo, Godwin Twakiire, Alain Favina, Gideon Munaru, Godfrey Zari Rukundo

**Affiliations:** 1 School of medicine, King Ceasor University, Kampala, Uganda; 2 Department of Psychiatry, Uganda Christian University, Kampala, Uganda; 3 African Center for Suicide Prevention and Research, Mbarara, Uganda; 4 Department of Psychiatry, Mbarara University of Science and Technology, Mbarara, Uganda

**Keywords:** Adolescents, Emotional and Behavioral difficulties, Body image and eating distress, Uganda

## Abstract

**Background:**

Adolescents frequently have emotional and behavioral difficulties as they struggle with the challenges of transition from childhood to adulthood. Many struggle with issues of body image and eating distress as they deal with the difficult and frequently perplexing changes that occur with puberty. Yet there is surprisingly little research on the emotional and behavioral challenges, as well as body image and eating distress among this sizable population in Uganda. This study sought to assess attitudes and behaviors related to body image and eating distress, as well as emotional and behavioral difficulties among adolescents in Mbarara, Southwestern Uganda.

**Methods:**

This was a cross-sectional study among 788 adolescents aged 13 to 19 years in secondary schools in Mbarara city and Mbarara district in south-western Uganda. The study employed the Body Image and Eating Distress scale to assess attitudes and behaviors about dieting and body shape and the extended version of the Strengths and Difficulties Questionnaire (SDQ) to assess for perceived emotional and behavioral difficulties. Logistic regression was used to identify the association between body image and eating distress and perceived difficulties.

**Results:**

The prevalence of high body image and eating distress was 10.8% while that of perceived emotional and behavioral difficulties was 45.8%. Some of the adolescents (16.1%) were dissatisfied with their body shape, 24.6% exercised a lot to avoid gaining weight, 15.0% were terrified to gain even a little weight, and 12.1% could not control their eating. More males reported eating large amounts of food at one time (*p* =  < 0.001). Having emotional and behavioral difficulties (aOR: 1.89; 95% CI: 1.18 – 3.02; *p* = 0.019) and coming from a two-parent household (aOR: 1.79; 95% CI: 1.10 – 2.92; *p* = 0.019) increased the odds of high body image and eating distress.

**Conclusion:**

High levels of body image and eating distress are linked to behavioral and emotional problems and adolescent’s family structure. Clinicians who treat adolescents should use a holistic care strategy and be aware of the high prevalence and close association between emotional and behavioral difficulties, concerns about weight, and dieting. It is important to encourage parental involvement and support in providing information about mental health issues among adolescents.

## Introduction

Body image and eating distress which encompasses a range of negative emotions related to food, eating, and negative body image is common among adolescents [1, 2]. It can include symptoms of disordered eating such as binge eating, purging, or restrictive eating patterns [1]. Many adolescents struggle with their body image as they deal with the difficult and frequently perplexing changes that occur with puberty [3, 4]. Physical and hormonal changes that accompany the shift from childhood to adolescence cause individuals to become more self-aware and compare themselves to others, which makes them feel insecure and dissatisfied with their bodies [1]. Adolescents frequently struggle with body acceptance and attempt to fit in with cultural expectations and harmful gender norms of beauty [5]. The ideal is for males to be thin and strong while girls should be slim and shapely, according to modern Western society that has significant influence on adolescents worldwide [5, 6]. Media representations and capitalism often perpetuate these unrealistic ideals, fostering feelings of inadequacy and dissatisfaction with one's appearance, which can contribute to the development or exacerbation of body image and eating distress [7, 8]. Yet, adolescent internet and social media use has skyrocketed in recent years [9]. Moreso than ever before, adolescents are more visible online today [10] with many adolescents owning devices having internet connection [11].

Adolescents who have unfavorable body image perceptions and eating distress are more likely to experience low self-esteem, anxiety, and even mental health problems [12]. They might develop eating disorders, such as anorexia and bulimia, as a way to cope with negative emotions and perceptions about food and body image. However, these disordered behaviors not only worsen their body image and eating distress but are also linked to health risks like stunted physical growth [13], irregular menstruation [14], physical weakness [15], chronic irritation [15], constipation [16], poor focus, trouble sleeping [17], depression [18], and substance abuse [19]. Moreover, the societal pressure to conform to unrealistic beauty standards and gender norms can exacerbate both mental health issues and body image/eating distress [5]. Various studies among adolescents have reported a prevalence of body image and eating distress in this demographic ranging from, 5% to 10% [20, 21]. Another study reported a prevalence of as high as 35% among female high school students [22].

Rapid physical, cognitive, and emotional changes that are characteristic of this stage of life can lead to increased emotional sensitivity and mood swings that cause emotional and behavioral difficulties [23, 24]. Adolescents frequently have emotional and behavioral difficulties as they struggle with the challenges of making the transition from childhood to adulthood [24]. Adolescents who are often trying to find their independence and figure out who they are may feel confused, irritable, and overwhelmed by their emotions [25]. Additionally, they could experience a variety of stresses like peer pressure, family problems, academic pressure, or societal expectations, which can exacerbate emotional and behavioral difficulties [26]. When dealing with these difficulties, some adolescents may display behaviors like rebellion, risk-taking, withdrawal, or aggressiveness [27].

Adolescents' emotional and behavioral difficulties have a strong and frequently entwined relationship with eating distress and their body image [28–30]. Adolescents who have eating distress and trouble with their body image are more prone to have emotional and behavioral problems [29]. The emotional and behavioral difficulties brought on by body image issues can significantly affect an adolescent’s general wellbeing resulting into poor quality of life [31]. Having a poor mental health outcome and a higher chance of adopting unhealthy coping methods like substance addiction or self-harm can result from having a bad body image, which can start a loop of negative thoughts and emotions [32]. Additionally, it might affect how well they perform academically and impede social growth since vulnerable adolescents may avoid social situations and opportunities [33].

Despite the recognized severity of emotional and behavioral challenges as well as body image and eating distress among this age group [31], there remains a scarcity of research addressing the emotional and behavioral hurdles, alongside concerns related to body image and eating distress, encountered by this population in Uganda. This obvious study vacuum is alarming, given that adolescence is a vital developmental stage that might affect a person's long-term wellbeing. This study therefore sought to assess attitudes and behaviors related to body image and eating distress, as well as emotional and behavioral difficulties among adolescents in Southwestern Ugandan. Findings from this study make it possible to identify the extent of the problem in order to create effective support networks and focused interventions to deal with these problems.

## Methods

### Study design and setting

This was a cross-sectional analytical study among adolescents in secondary schools in Mbarara city and Mbarara district in southwestern Uganda. Mbarara city has 37 secondary schools (9 government, 28 private). The district has 14 public and 24 privately owned secondary schools [34], and the estimated overall population of learners in secondary schools in Mbarara city and Mbarara district is about 20,259 and 14,882 respectively. As with the rest of Uganda, secondary education in southwestern Uganda, is divided into ordinary level (O’ level) and the advanced level (A’ level) [35]. The ordinary level provides schooling for mostly the age group of 13–16 years of age and takes four school years from senior-one class to senior-four class. The advanced level of secondary education provides education for the age group of roughly 17–19 years and takes two school years from senior-five class to senior-six class [36]. At the end of the ordinary level education, one qualifies for Uganda Certificate of Education (UCE) while at the end of advanced level, one qualifies for Uganda Advanced Certificate of Education (UACE). The secondary schools are either government-owned/aided (public) or privately-owned and can either be single-sex schools or mixed-sex schools [36]. We selected 10 schools whose Heads provided administrative clearance Half of the schools were public. Three of the schools were located in a rural setting and others in urban setting and four of the schools were single-sex schools.

### Study population and eligibility screening

The target population for this study was adolescents (13–19 years). A total of 788 individuals agreed to participate in the study. We excluded individuals who were physically incapable of completing the interview due to illness; those who were below 18 years whose parents did not consent and those who did not ascent. We also excluded those who were 18 and 19 years who did not consent to participate.

### Data collection

The data were collected by four research assistants who were trained in data collection, research ethics and questionnaire administration. The potential participants were chosen and enrolled after completing all the procedures involving administrative clearance from the school heads; consent from parents and ascent of those below 18 years; and consent from those 18 and 19 years of age. Each participant was given a unique identification number to maintain anonymity. The surveys were completed in a controlled classroom setting, with guaranteed privacy and confidentiality.

### Study tools and measures

This study utilized a self-administered questionnaire, originally used in a survey conducted in Finland by Koskelainen et al. [3]. The questionnaire included socio-demographic information such as age, sex, family structure (two biological parents versus single biological parent versus other family head), weight, height, body image and eating distress symptoms and perceived emotional, concentration and behavioral difficulties. It took about 30–45 min to complete the entire questionnaire. On the basis of measured height and weight, respondents were categorized in as underweight (BMI less than 18.5), normal weight (BMI of 18.5 to less than 25), overweight (BMI of 25 to less than 30) and obese (BMI of 30 or higher). Social economic status of the family was assessed using the question “How well do you think your family is in relation to other families, regarding economic situations?” and responses were categorized as low social economic status (if response was “not good”); moderate social economic status (if response was “relatively good or averagely good or somewhat good”) and high social economic status (if response was “very good”).

### Body image and eating distress scale

To assess attitudes and behaviors about dieting and body shape, a brief-item "Body Image and Eating Distress" scale created by Koskelainen, Sourander & Helenius [3, 5] was employed (Table 1). The statements were given a score of zero if they were untrue, one if they were partially true, and two if they were definitely true. The overall score for eating disorders, which ranged from 0 to 18, was determined by adding the items together. A high level of body image and eating distress was denoted by the 90th percentile cut-off point of the scoring distribution on the total eating disturbance scale [3].

**Table 1:** Characteristics and distribution of level of body image and eating distress and difficulties in areas of either emotions, concentration, behavior, or getting along with others

**Variable** *n* = 788	**n (%)**	**Body image and eating distress**		**X**^**2**^** /t (*p*-value)**	**Difficulties**		**X**^**2**^**/t (*p*-value)**
		**Low** 703 (89.2%)	**High** 85 (10.8%)		**No** 427 (54.2%)	**Yes** 361 (45.8%)	
Age (mean/SD)	16.6 (1.9)	16.5 (1.8)	17.0 (2.0)	**-2.08 (0.037)**	16.5 (1.8)	16.7 (2.0)	-1.44 (0.253)
**Sex**							
Female	320 (40.6)	288 (90.0)	32 (10.0)	0.35 (0.556)	167 (52.2)	153 (47.8)	0.87 (0.351)
Male	468 (59.4)	415 (88.7)	53 (11.3)		260 (55.6)	208 (44.4)	
**Class**							
O’ level	664 (84.3)	593 (89.3)	73 (10.7)	0.04 (0.844)	350 (52.7)	314 (47.3)	3.71 (0.054)
A’ level	124 (15.7)	110 (88.7)	14 (11.3)		77 (62.1)	47 (37.9)	
**Family social economic status** (*rating of one’s family compared to other families*)							
Low	121 (15.3)	103 (85.1)	18 (14.9)	3.47 (0.177)	60 (49.6)	61 (50.4)	2.10 (0.350)
Moderate	502 (63.7)	455 (90.6)	47 (9.4)		271 (54.0)	231 (46.0)	
High	165 (21.0)	145 (87.9)	20 (12.1)		96 (58.2)	69 (41.8)	
**Family structure**							
Two – parent family (*including biological and step-parents*)	320 (40.6)	275 (85.9)	45 (14.1)	**6.01 (0.014)**	166 (51.9)	154 (48.1)	1.16 (0.281)
Other (*including single-parent family and those headed by other relatives*)	468 (59.4)	428 (91.5)	40 (8.5)		261 (55.8)	207 (44.2)	
**BMI Category**							
Underweight	76 (9.6)	69 (90.8)	7 (9.2)	0.51 (0.916)	38 (50.0)	38 (50.0)	3.99 (0.263)
Normal	642 (81.5)	571 (88.9)	71 (11.1)		358 (55.8)	284 (44.2)	
Overweight	55 (7.0)	50 (90.9)	5 (9.1)		24 (43.6)	31 (56.4)	
Obese	15 (1.9)	13 (86.7)	2 (13.3)		7 (46.7)	8 (53.3)	

*SD* Standard Deviation

### The extended version of the strengths and difficulties questionnaire

The extended version of the Strengths and difficulties questionnaire (SDQ) developed by Goodman [37] was used to assess perceived emotional, concentration and behavioral difficulties among the adolescents. It includes an impact supplement that asks if the respondent thinks the young person has a problem, and if so, enquires further about chronicity, distress, social impairment, and burden for others [37]. The tool has been used in other countries in Sub-Saharan Africa [38, 39]. Individuals who responded affirmatively to the question, "In general, do you think you have problems in any of the following areas: emotions, concentration, behavior, or getting along with others?" were categorized as having emotional, concentration, and behavioral difficulties. Subsequently, those who answered "yes" proceeded to answer additional inquiries, including "How long have you been experiencing these problems?" and "Do these problems make you sad or upset?". The impact of these problems on daily life was evaluated through the question, "Do these problems interfere with your daily life in the areas of (i) Home life (routine life in the home), (ii) Friendships, (iii) Classroom learning, (iv) Leisure activities?", with responses coded as follows: 0 for "No", 1 for "Very little", 2 for "To a great extent", and 3 for "Very much". Furthermore, participants were asked, "Do these problems make it harder for those around you (family, friends, teachers, etc.)?" We opted against utilizing the total difficulties score or subscale scores from the SDQ due to our emphasis on simplicity and conciseness in data collection. Our aim was to bolster participant engagement and reduce respondent burden, taking into account factors such as time constraints.

### Ethical considerations

The study was conducted in adherence to the ethical principles outlined in the 2013 Declaration of Helsinki criteria. The research protocol was approved by the research ethics committee of Mbarara University of Science and Technology (MUST-2022–673). Consent from parents for students aged below 18 years was obtained. Prior to enrollment in the study, all participants provided written informed consent or ascent voluntarily, after being fully informed of the study's objectives and procedures. The school administrators authorized the collection of data from the participants. Before conducting the study, the research team received training on identifying individuals with severe psychological distress. Individuals found to have severe psychological distress were referred for to the school counsellors who were trained and assisted by the psychiatrists on the research team for counseling before continuing with the study. If they encountered psychological distress again during the study, their participation was halted, and they were referred to the school counselor for additional support services.

### Data analysis

The data were cleaned and analyzed using STATA version 16 software. Descriptive statistics such as mean, mode, median, and standard deviations were used to summarize continuous variables, while proportions and percentages were used to describe categorical variables. Pearson's chi-square test was employed to compare categorical data between the two groups. The independent sample t-test compare numerical variables and Mann–Whitney U test were used to compare two independent samples when data did not meet the assumptions of parametric tests. To determine the association between body image and eating distress and emotional and behavioral difficulties, logistic regression models were used and social demographic characteristics were controlled for. All statistical analyses were performed with a 95% level of confidence and a 5% margin of error.

## Results

### Characteristics of participants

The average age of all 778 participants was 16.6 ± 1.9 years, most (59.4%, *n* = 468) were male, in in ordinary level (O’ level) (84.3%, *n* = 664) and were living with relatives other than their parents (46.6%, *n* = 363) other than their biological parents (See Table 1).

### Distribution of level of body image and eating distress and difficulties in areas of either emotions, concentration, behavior, or getting along with others

The proportion of individuals with high levels of body image and eating distress was 10.8%, *n* = 85. Those who had high levels of body image and eating distress were older than those with low levels (mean age of 17.0 ± 2.0 vs 16.5 ± 1.8, *p* = 0.028). More adolescents living in a two-parent household had high levels of body image and eating distress (**X**^**2**^ = 8.08, *p* = 0.018).

The proportion on individual with emotional and behavioral difficulties was 45.8%, *n* = 361. (see Table 1).

### Body image and eating distress items across gender

Some adolescents (30.3%) were afraid of becoming fat; 14.3% wanted to be thinner, 19.8% were on a diet, 24.6% exercised a lot to avoid gaining weight, 16.1% were dissatisfied with their body shape, 19.0% lost weight in a short time due to not eating, 15.0% were terrified to gain even a little weight, and 12.1% could not control their eating. More females reported losing weight in a short time due to not eating than males (26.1% vs. 13.9%, **X**^**2**^ = 20.79, *p* =  < 0.001) while more males reported eating large amounts of food at one time than females (8.1% vs. 3.1%, **X**^**2**^ = 22.65,* p* =  < 0.001) (Table 2).

**Table 2:** Perceptual response rates of the body image and eating distress items across gender

**Variable**	**n (%)** *n* = 788	**Gender**		**X**^**2**^** (*p*-value)**
		**Female** 320 (40.6%)	**Male** 468 (59.4%)	
**Want to be thinner**				
Not true	572 (72.6)	235 (73.4)	337 (72.0)	0.36 (0.835)
Somewhat true	103 (13.1)	42 (13.1)	61 (13.0)	
Certainly true	113 (14.3)	43 (13.4)	70 (15.0)	
**On a diet**				
Not true	427 (54.2)	165 (51.6)	262 (56.0)	2.24 (0.327)
Somewhat true	205 (26.0)	92 (28.8)	113 (24.1)	
Certainly true	156 (19.8)	63 (19.6)	93 (19.9)	
**Exercise a lot to avoid gaining weight**				
Not true	379 (48.1)	159 (49.7)	220 (47.0)	2.75 (0.252)
Somewhat true	215 (27.3)	92 (28.8)	123 (26.3)	
Certainly true	194 (24.6)	69 (21.6)	125 (26.7)	
**Afraid of getting fat**				
Not true	380 (48.2)	166 (51.8)	214 (45.7)	5.05 (0.080)
Somewhat true	169 (21.5)	71 (22.2)	98 (20.9)	
Certainly true	239 (30.3)	83 (25.9)	156 (33.3)	
**Lost weight in a short time due to not eating**				
Not true	505 (64.1)	181 (56.6)	324 (69.2)	**20.79 (< 0.001)**
Somewhat true	133 (16.9)	54 (16.9)	79 (16.9)	
Certainly true	150 (19.0)	85 (26.1)	65 (13.9)	
**Dissatisfaction with body shape**				
Not true	522 (66.2)	203 (63.4)	319 (68.2)	1.90 (0.387)
Somewhat true	139 (17.6)	61 (19.1)	78 (16.7)	
Certainly true	127 (16.1)	56 (17.5)	71 (15.2)	
**Terrified to gain even a little weight**				
Not true	527 (66.9)	212 (66.2)	315 (67.3)	0.40 (0.819)
Somewhat true	143 (18.1)	57 (17.8)	86 (18.4)	
Certainly true	118 (15.0)	51 (15.9)	67 (14.3)	
**Cannot control eating**				
Not true	587 (74.5)	240 (75.0)	347 (74.1)	0.34 (0.842)
Somewhat true	106 (13.4)	44 (13.7)	62 (13.3)	
Certainly true	96 (12.1)	36 (11.3)	59 (12.6)	
**Devour large amounts of food at one time**				
Not true	681 (86.4)	299 (93.4)	382 (81.6)	**22.65 (< 0.001)**
Somewhat true	59 (7.5)	11 (3.4)	48 (10.3)	
Certainly true	48 (6.1)	10 (3.1)	38 (8.1)	
**Purposely vomit after eating**				
Not true	744 (94.4)	302 (94.4)	442 (94.4)	0.86 (0.649)
Somewhat true	28 (3.5)	10 (3.1)	18 (3.8)	
Certainly true	16 (2.0)	8 (2.5)	8 (1.7)	
**Used medications to control my weight (appetite suppressants, laxatives, or diuretics)**				
Not true	721 (91.5)	287 (89.7)	434 (92.7)	3.01 (0.221)
Somewhat true	36 (4.5)	16 (5.0)	20 (4.3)	
Certainly true	31 (3.9)	17 (5.3)	14 (3.0)	

### Relationship between emotional and behavioral difficulties, body image and eating distress

More individuals with emotional and behavioral difficulties had high body image and eating distress (60.0% vs 44.1%, X^2^ = 7.73, *p* = 0.005). Adolescents with high body image and eating distress statistically had more negative impact of the difficulties on their lives (in areas of home life, friendships, classroom learning or leisure activities) than those low body image and eating distress i.e., median of 2 vs 0, respectively, *p* value < 0.001. Additionally, more individuals who reported that these difficulties only made it a lot harder for others had high body image and eating distress (X^2^ = 19.50, *p* =  < 0.001) (Table 3).

**Table 3:** Body image and eating distress and Difficulties in areas of either emotions, concentration, behavior, or getting along with others

**Variable**	n (%)	**Body image and eating distress**		**X**^**2**^** t (*p*-value)**
		**Low** 703 (89.2%)	**High** 85 (10.8%)	
**Difficulties**				
No	427 (54.2%)	393 (55.9)	34 (40.0)	7.73 (0.005)
Yes	361 (45.8%)	310 (44.1)	51 (60.0)	
**Duration of experience of difficulties**				
Less than a month	162 (44.1)	138 (43.9)	24 (45.3)	0.80 (0.850)
1 – 5 months	45 (12.4)	37 (11.8)	8 (15.1)	
6 – 12 months	40 (11.1)	34 (10.8)	6 (11.3)	
More than a year	120 (32.4)	105 (33.4)	15 (28.2)	
**Impact of the difficulties on one’s life** (Median/ IQR)	0 (0—2)	0 (0—2)	2 (0—4)	**-4.95 (< 0.001)**
**Difficulties make it harder for others** (family, friends, teachers etc.)				
A little	707 (89.7)	642 (91.3)	65 (76.5)	**19.50 (< 0.001)**
Averagely	79 (10.0)	60 (8.5)	19 (22.3)	
A lot	2 (0.2)	1 (0.1)	1 (1.2)	

*IQR* Interquartile range

### Association between emotional and behavioral difficulties and high body image and eating distress

Multivariate logistic regression was carried out while controlling for all social demographic variables. These first were tested for collinearity; all the factors had a VIF below two and mean VIF of 1.15. Having emotional and behavioral difficulties increased odds of having high body image and eating distress (aOR: 1.84; 95% CI: 1.15 – 2.95; *p* = 0.011) (Table 4).

**Table 4:** Association between difficulties in areas of either emotions, concentration, behavior, or getting along with others and body image and eating distress

Variable	High body image and eating distress			
	**Crude Odds Ratio/ cOR (95% confidence interval/ CI)**	***p*-value**	**Adjusted Odds Ratio/ aOR (95% confidence interval/ CI)**	***p*-value**
**Emotional and behavioral Difficulties**				
No	1 (Reference)	0.006	1 (Reference)	
Yes	1.90 (1.20—3.00)		1.84 (1.15 – 2.95)	**0.011**
**Age**	1.14 (1.01 – 1.30)	0.039	1.13 (0.98 – 1.31)	0.097
**Sex**				
Female	1 (Reference)		1 (Reference)	
Male	1.15 (0.72 – 1.83)	0.556	1.27 (0.78 – 2.08)	0.334
**Class**				
O’ level	1 (Reference)		1 (Reference)	
A’ level	1.06 (0.58 – 1.95)	0.844	1.19 (0.57 – 2.48)	0.650
**Family social economic status***(rating of one’s family compared to other families)*				
Low	1 (Reference)		1 (Reference)	
Moderate	0.59 (0.32 – 1.05)	0.078	0.72 (0.38 – 1.32)	0.290
High	0.79 (0.40 – 1.57)	0.498	0.95 (0.46 – 1.98)	0.903
**Parent lived with in past month**				
Other-relative headed family	1 (Reference)		1 (Reference)	
Single-parent family	1.76 (0.87 – 3.54)	0.114	1.64 (0.78 – 3.47)	0.192
Two-parent family	2.04 (1.23 – 3.37)	0.006	2.09 (1.21 – 3.65)	**0.009**
**BMI Category**				
Underweight	1 (Reference)		1 (Reference)	
Normal	1.22 (0.54 – 2.77)	0.625	1.27 (0.54 – 2.95)	0.619
Overweight	0.99 (0.29 – 3.29)	0.981	0.84 (0.24 – 2.92)	0.767
Obese	1.51 (0.28 – 8.13)	0.627	1.03 (0.18 – 5.60)	0.982

### Other factors associated with high body image and eating distress

Living in a two-parent household increased odds of having high body image and eating distress (aOR: 2.09; 95% CI: 1.21 – 2.65; *p* = 0.009) (Table 4).

## Discussion

We aimed to determine the association between body image and eating distress and emotional- behavioral difficulties among school going adolescents in Uganda. According to our findings, the prevalence of high body image and eating distress was 10.8% and 45.8% for emotional and behavioral difficulties, among adolescents in Uganda. More adolescents with emotional and behavioral difficulties had statistically significantly higher body image and eating distress. Those who had more negative impact of the difficulties on their lives (in areas of home life, friendships, classroom learning or leisure activities) had statistically significantly higher body image and eating distress. Additionally, emotional and behavioral difficulties and living in a two-parent household increased the odds of having high body image and eating distress.

The high prevalence of body image and eating distress among adolescents in this study aligns with similar studies conducted in different parts of the world, indicating that body image and eating disorders are not limited to specific regions or cultures [20, 21, 40]. It sheds light on an important issue affecting this population in Uganda. Urgent attention is needed to prevent the escalation of these problems into more severe eating disorders and associated health risks. Interventions focused on fostering positive body image, encouraging healthy eating habits, and teaching effective coping mechanisms are essential for mitigating the adverse effects of body image and eating distress in adolescents, thereby enhancing their long-term health prospects.

More females in this study reported losing weight in a short time due to not eating while more males reported eating large amounts of food at one time. This finding provides valuable insights into gender differences in eating behaviors and weight control strategies among adolescents. It aligns with a study in the United States of America (USA) that showed females tend to report higher rates of restrictive eating, dieting, and weight concerns compared to males [40].

On the other hand, the finding that more males in this study reported eating large amounts of food at one time is inconsistent with observations from a study among adolescents in USA that showed higher rates of binge eating behaviors among females [41]. The difference in the findings is likely due to cultural differences in the study areas and possibly due differences in study tools. Additionally, lack of validation of the questionnaire use in the current context might have contributed to the different findings.

The finding in this study that eating distress and negative body image was statistically higher among older adolescents compared to the younger ones aligns with the broader patterns observed in numerous studies examining the relationship between age and body image concerns [42, 43].

The high prevalence of emotional and behavioral difficulties raises serious concerns regarding the mental health situation in the country. This data highlights the pressing need for tailored interventions and support networks to address the obstacles encountered by young individuals. Various factors contribute to this concerning prevalence rate. One crucial element is the socio-economic context prevalent in Uganda, characterized by widespread poverty, limited access to quality education and healthcare, and exposure to violence and trauma [44]. These challenges can profoundly impact the mental well-being of adolescents, intensifying feelings of stress, anxiety, and depression. Additionally, the stigma attached to mental illness may deter adolescents from seeking assistance or receiving adequate support from their communities and families. Furthermore, there may be a lack of awareness about mental health issues and available resources, further obstructing access to care [45].The prevalence of emotional and behavioral difficulties (45.8%) among adolescents in this study was higher than 11.7% and 10.3% among Jordanian adolescents [46] and school-going adolescents in India, respectively [47]. The differences in the findings are likely because unlike the studies in Jordan and India, this study based on self-report which relies on individuals' own perceptions and interpretations of their emotional and behavioral difficulties rather than symptoms and can lead to a higher prevalence as individuals may report a wider range of difficulties based on their own understanding and experiences [48].

This study discovered several key findings: (i) Individuals with emotional and behavioral difficulties experienced higher levels of body image and eating distress; (ii) Those who reported greater negative impacts of these difficulties on various aspects of their lives, such as home life, friendships, classroom learning, or leisure activities, also exhibited higher levels of body image and eating distress. These findings are consistent with previous research conducted among adolescents in Finland and Japan [3, 5]; which explain that negative body image and disordered eating behaviors might serve as maladaptive coping mechanisms disordered eating to deal with emotional distress [3, 5].

The finding in this study that living in two-parent households increased the odds of having high body image and eating distress is likely because this family environment may provide more financial stability and resources, which could lead to an emphasis on appearance and the ability to afford beauty-related products that may contribute to unrealistic beauty standards [49, 50].

Our study findings emphasize the need for improved adolescent mental health services accessibility and availability, early detection and intervention for problems with emotional and behavioral difficulties, as well as food and body image discomfort. The results highlight the importance of taking a comprehensive approach to managing adolescents' health issues by understanding how numerous factors are interconnected. The findings of this study indicate that there is a need to promote parental participation and support while also raising awareness of and education about mental health issues among adolescents. The prevalence of eating distress body-image problems among adolescents highlights the need for preventive treatments that encourage age-appropriate eating and dieting habits.

### Strengths and limitations of the study

This study included a number of limitations that should be taken into account. The participants' characteristics may not be indicative of the overall population given that the study sample was limited to a single geographic location in Uganda but included students from boarding schools who came from all over the nation. Second, although the tools used in the study demonstrated acceptable reliability, the "Body image and Eating Distress" questionnaire had not been validated using clinical samples, raising the possibility of misinterpretation or cultural differences in how respondents interpreted questions about body image and eating distress. Furthermore, it is always feasible that subjects reporting distress and behavioral issues may differ between cultures. Third, because the research was cross-sectional and dependent on self-reports, it was unable to determine the cause and effect. Therefore, prospective and qualitative studies are advised. The questionnaire focused on attitudes and behaviors related to dieting and body shape, although some of the items were strikingly similar to those found, for example, in the Eating Disorders Inventory [38], a considerably longer but thoroughly validated questionnaire. It's critical to recognize that diets and body image issues affect adolescent wellbeing more broadly than just the clinical diagnoses [5]. Furthermore, there was a potential of missed opportunities for thorough assessment and nuanced analysis when not utilizing the total difficulties score or subscale scores from the SDQ. Additionally, although it's feasible to make broad conclusions about the links between Social Economic Status and body image and eating distress, it's important to acknowledge a potential constraint that the measure used to evaluate SES was relatively weak, relying on a single non-specific and subjective question. Consequently, the analysis was more exploratory and the results mainly highlight general patterns derived from the analysis, indicating the necessity for a more rigorous study.

## Conclusions

The study highlights the significant burden of eating and body image-related distress and that of emotional and behavioral difficulties among adolescents in Uganda. Emotional and behavioral difficulties and coming from a two-parent household are associated with high levels of concern with food and body image. Clinicians who evaluate or treat adolescents should be aware of the high prevalence and the strong link between emotional and behavioral issues and worries about weight and dieting and take a holistic approach to management. In addition to raising awareness of and providing information about mental health issues among adolescents, it is important to encourage parental involvement and support.

## Data Availability

The datasets related to this manuscript is available under the following DOI; 10.6084/m9.figshare.25641078.

## Abbreviations

SDQ: Strengths and Difficulties Questionnaire
BMI: Body Mass Index
O’ level: Ordinary level
A’ level: Advanced level
